# High-throughput Production of ZnO-MoS_2_-Graphene Heterostructures for Highly Efficient Photocatalytic Hydrogen Evolution

**DOI:** 10.3390/ma12142233

**Published:** 2019-07-11

**Authors:** Haocong Dong, Junzhu Li, Mingguang Chen, Hongwei Wang, Xiaochuan Jiang, Yongguang Xiao, Bo Tian, Xixiang Zhang

**Affiliations:** 1 Key Laboratory of Film Materials & Application for Equipment, School of Materials Science and Engineering, Xiangtan University, Xiangtan 411105, China; 2 Eleven-Dimensional Nanomaterial Research Institute, Xiamen 361000, China; 3 Physical Science and Engineering Division, King Abdullah University of Science and Technology (KAUST), Thuwal 23955-6900, Saudi Arabia; 4 State Key Laboratory of Advanced Optical Communication Systems and Networks, Department of Electronic Engineering Shanghai Jiao Tong University, Shanghai 200240, China; 5 Department of Astronomy, Xiamen University, Xiamen 361000, China

**Keywords:** graphene, MoS_2_, ZnO, photocatalyst, high-throughput production

## Abstract

High-throughput production of highly efficient photocatalysts for hydrogen evolution remains a considerable challenge for materials scientists. Here, we produced extremely uniform high-quality graphene and molybdenum disulfide (MoS_2_) nanoplatelets through the electrochemical-assisted liquid-phase exfoliation, out of which we subsequently fabricated MoS_2_/graphene van der Waals heterostructures. Ultimately, zinc oxide (ZnO) nanoparticles were deposited into these two-dimensional heterostructures to produce an artificial ZnO/MoS_2_/graphene nanocomposite. This new composite experimentally exhibited an excellent photocatalytic efficiency in hydrogen evolution under the sunlight illumination (λ>400 nm), owing to the extremely high electron mobilities in graphene nanoplatelets and the significant visible-light absorptions of MoS_2_. Moreover, due to the synergistic effects in MoS_2_ and graphene, the lifetime of excited carriers increased dramatically, which considerably improved the photocatalytic efficiency of the ZnO/MoS_2_/graphene heterostructure. We conclude that the novel artificial heterostructure presented here shows great potential for the high-efficient photocatalytic hydrogen generation and the high throughput production of visible-light photocatalysts for industrial applications.

## 1. Introduction

Photocatalytic hydrogen-evolution nanotechnologies under visible-light illumination have attracted tremendous and growing interest from the scientific communities due to their significant potential for the storage of solar energy and the production of green energy. In recent decades, various methods and nanostructures have been developed to realize efficient hydrogen generation [1,2]. As examples, a photostable photocatalyst of n-doped zinc oxide (ZnO)/graphene oxide has been developed for improving the mineralization and photodegradation of organic dye under visible light [3,4,5]; ‘‘green” SnS_2_ quantum dots/reduced graphene oxide composites have been fabricated with enhanced photocatalytic performance [6]; and Pd-decorated ZnO−graphene oxide nanocomposites have been reported with significantly enhanced photocatalytic activities based on a charge separation mechanism [7]. However, due to the limited lifetime of excited electrons and holes, as well as the poor abilities of visible-light absorption in ordinary materials, high-efficient mass-produced sunlight-driven photocatalysts remain strongly desired.

Low-dimensional nanomaterials, such as quantum dots and two-dimensional materials, are expected to be the new-generation photocatalytic materials because of their excellent physical and chemical properties. Zinc oxide (ZnO) nanoparticles are emerging as a competitive photocatalyst, due to their outstanding photoelectricity, regulable band gap, and extremely high electron densities [8,9]. However, the rapid recombination of photogenerated electrons and holes in ZnO nanoparticles, as well as the poor capabilities of visible-light absorption deteriorated the ZnO nanoparticles’ photocatalytic performances [10,11,12,13]. Even though some researchers have managed to combine the ZnO nanoparticles with graphene oxide [14], which served as the electron transport layer, the photocatalytic efficiency was not significantly improved, because it was restricted by the self-aggregation effect of these nanoparticles and the low conductivity of graphene oxide. 

Graphene, the ‘superstar’ of two-dimensional materials with excellent electric, thermal, and mechanical properties [15,16,17], was used to replace graphene oxide in order to improve the photocatalytic performance of ZnO/graphene oxide heterostructures by reducing the recombination rates of excited electrons and holes in the photocatalytic processes [18,19,20,21,22,23]. However, ZnO/graphene structures still could not reach a satisfactory catalytic performance, due to the weak absorption capability of visible light. For this reason, we proposed to fabricate a ZnO/MoS_2_/graphene heterostructure, in which MoS_2_, generally considered to be a layered n-type semiconductor with a suitable band gap for the absorption of visible light, serves as an interlayer to the sunlight absorption and thus improves the photocatalytic efficiency [24,25,26]. In addition, this two-dimensional MoS_2_ also significantly enhances the transportation of excited carriers in the composite, leading to a higher photocatalytic efficiency [27,28].

In this work, we exfoliated graphite into high-quality graphene nanoplatelets in solvents, produced high-quality layered MoS_2_ by the electrochemical-assisted liquid-phase exfoliation method [29], and assembled these two-dimensional materials to MoS_2_/graphene van der Waals heterostructures [30]. Next, ZnO nanoparticles were chemically synthesized and incorporated into the MoS_2_/graphene nanostructures to form ZnO/MoS_2_/graphene ternary heterostructures. Relying on the extremely-high carrier mobilities and the large effective surface of graphene nanoplates, as well as the enhanced visible-light absorption of the electron-transport-layered MoS_2_ [31,32,33,34], these heterostructures no only exhibited the high photocatalytic efficiency in the generation of hydrogen but also reached an efficiency five times higher than that previously reported of graphene-oxide-based photocatalysts. It is worth noting that the new heterostructures were quite easily synthesized, as the preparation process of non-oxidized pure graphene nanoplatelets is faster and more straightforward, owing to the lack of oxidation-reduction process [35]. Our synthetic process also offers the possibility to be performed in large-scale ultrasound machines, making it suitable for the large-scale industrial production of the ZnO/MoS_2_/graphene heterostructure.

## 2. Materials and Methods 

### 2.1. Preparation of Graphene Nanoplatelets

Graphene nanoplatelets were produced by dispersing expanded highly oriented pyrolytic graphite (HOPG; Aldrich product # 332461, Shanghai, China) in N-methyl-pyrrolidone (NMP; spectrophotometric 99.0%) solvent via bath sonication (100 kHz) for 10 h at 60 °C [36]. After that, the samples were centrifuged at 10,000 rpm for 1 h to extract the macroscopic aggregations. After repeating the sonication and centrifugation processes 3 times, a homogeneous gray solution was obtained. This liquid-phase exfoliation method was employed to obtain graphene solutions for various graphene concentrations (up to 2.5 mg ml^−1^). In order to evaluate the morphology and quality of these samples, a small quantity of diluted graphene solution was dropped on the 300 nm SiO_2_/Si wafer and dried for further characterizations by scanning electron microscopy (SEM) and Raman spectroscopy.

### 2.2. Preparation of MoS_2_ Nanoplatelets 

Similar to the preparation of graphene nanoplatelets, we prepared MoS_2_ nanoplatelets by electrochemical-assisted liquid-phase exfoliation methods. First, we pretreated the MoS_2_ crystals to be expanded to be bulk MoS_2_ by using the electrochemical method previously reported by Duan’s group [37]. The expanded MoS_2_ crystal was sonicated in the dimethylformamide (DMF) solvent with the stabilizing-agent Poly-vinylpyrrolidone (PVP; molecular weight of about 40,000, Sigma-Aldrich, Shanghai, China), which effectively minimized the re-stacking of the MoS_2_ nanoplatelets for 6 h to obtain the MoS_2_ nanoplatelets. After the sonication and centrifugation processes were repeated, various concentrations (up to around 5 mg ml^−1^) of MoS_2_ solutions were produced. The solution exhibited a uniform yellow color due to the strong absorption of visible light by MoS_2_. The SEM and Raman spectroscopy data were collected by dropping the MoS_2_ nanoplatelets onto the surface of a 90 nm SiO_2_/Si wafer.

### 2.3. Preparation of ZnO Nanoparticles

In the process of synthesizing ZnO nanoparticles, 22 mg of zinc acetate dihydrate (Zn(CH_3_COO)_2_·2H_2_O; 99.999%, Sigma-Aldrich, Shanghai, China) was refluxed and dissolved in 10 mL ethanol at 80 °C. A potassium hydroxide solution, prepared from 95 mg of potassium hydroxide (KOH; ≥ 85%, Sigma-Aldrich, Shanghai, China) sonicated in 10 ml ethanol, was slowly added dropwise to the zinc acetate solution in a water bath at 50 °C under magnetic stirring. The reaction conditions were maintained for 1 h, and the resulting transparent solution was kept overnight, extracted with n-hexane and ethanol, washed, and air-dried. 

### 2.4. Assemblage of ZnO/MoS_2_/Graphene Heterostructures

**Figure 1:**
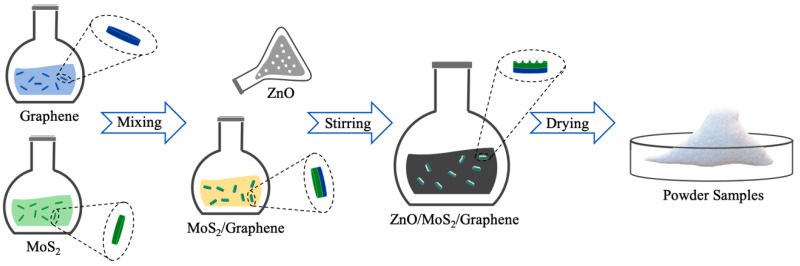
Schematics of manufacture processes of zinc oxide (ZnO)/molybdenum disulfide (MoS_2_)/graphene heterostructures.

First, in order to assemble the MoS_2_/graphene van der Waals heterostructures, dried MoS_2_ and graphene nanoplatelets were mixed and added into deionized water for dispersion and gelatinization. After that, the mixture solution was centrifuged at 4000 rpm for 30 min and dried at 200 °C on the hot plate in order to obtain the MoS_2_/graphene nanocomposites. It is worth noting that there may be existing a small amount of self-stacking graphene and MoS_2_ nanoflakes in the composite. After the preparation of MoS_2_/graphene heterostructures, we mixed prefabricated ZnO nanoparticles with dried MoS_2_/graphene in 20 mL deionized water, using the magnetic stirrer (mass fraction: MoS_2_ ~7 wt %; graphene ~3 wt %; and ZnO ~90 wt %), to ultimately produce the ZnO/MoS_2_/graphene heterostructures shown in Figure 1.

### 2.5. Characterization of Materials

Raman spectra and mappings of graphene and MoS_2_ were obtained using confocal Raman spectroscopy (WITec Alpha 300R, Ulm, Germany) with a 488 nm laser wavelength, a 10 mW laser energy power, and a 300 nm resolution. The morphology of the nanomaterials was obtained by imaging using a scanning electron microscope (SEM; ZEISS MERLIN, Jena, Germany). The components were detected by x-act with INCA and Aztec energy-dispersive X-ray spectroscopy (EDS) analysis software (Oxford Instruments, London, UK). The morphology and size of ZnO nanoparticles were measured by transmission electron microscope (TEM; Titan Themis Z, Oregon, USA) with an accelerating voltage of 300 kV. The X-ray diffraction patterns of the samples were tested using an X-ray diffractometer (XRD; Bruker D8 ADVANCE, Karlsruhe, Germany) with Cu (Kα) at a speed of 2°/min. The structures were detected by the X-ray photoelectron spectrometer (XPS; Kratos AXIS Ultra DLD, Manchester, UK). The UV-visible spectra of the samples were recorded on a spectrophotometer (Thermo Evolution 600 UV-Vis, Atlanta, OR, USA).

### 2.6. Evaluation of Photocatalytic Activity 

Hydrogen-generation photocatalytic measurements were conducted in a homemade quartz reactor system. Photocatalysts were placed into the deionized water with sacrificial reagents of sodium sulfide (Na_2_S; Sigma-Aldrich #407410) and sodium sulfite (Na_2_SO_3_; ≥ 85%, Sigma-Aldrich). The resulting mixture was stirred for 1 h without light in order to obtain a uniform dispersion. In the meantime, the nitrogen gas was introduced to exhaust the oxygen of the reaction chamber. We exposed the Xenon lamp (300 W, MKS 300XF, Kentucky, MA, USA), with a 400 nm center-wavelength standard bandpass filter, continuously stirred the reactor, and then measured the light intensities by the optical sensor. Meanwhile, the generated hydrogen was collected and analyzed using gas chromatography (Shimadzu, Nexis SCD-2030, Kyoto, Japan).

## 3. Results

### 3.1. Morphology Characterization of Materials

**Figure 2:**
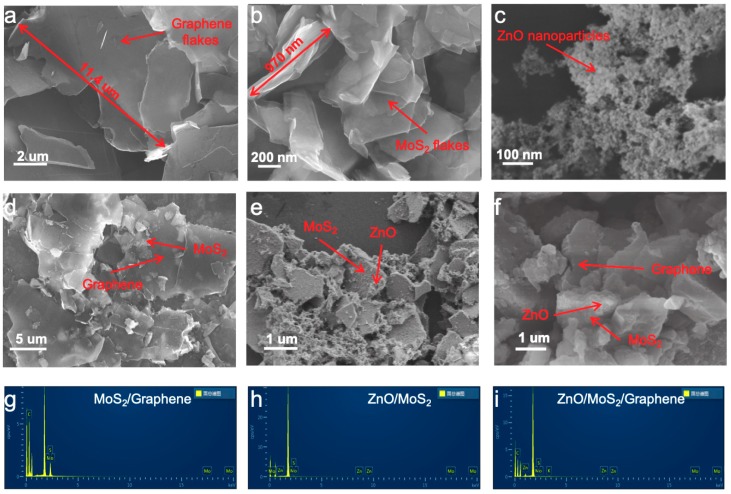
Morphology characteristics of the different samples. (**a**) SEM images of graphene nanoplatelets; (**b**) SEM images of MoS_2_ nanoplatelets; (**c**) SEM images of ZnO nanoparticles; (**d**) SEM images of MoS_2_/graphene; (**e**) SEM images of ZnO/MoS_2_, (**f**) SEM images of ZnO/MoS_2_/graphene; (**g**) energy-dispersive X-ray spectroscopy (EDS) of MoS_2_/graphene heterostructures; (**h**) EDS of ZnO/MoS_2_ heterostructures; (**i**) EDS of ZnO/MoS_2_/graphene ternary heterostructures.

The SEM characterization of the morphology of prepared MoS_2_ and graphene nanoplatelets are shown in Figure 2: Both of the prepared MoS_2_ and graphene exhibited layered structures can be seen in Figure 2a,b. It is evident that the graphene nanoplatelets exhibited an approximately 10 um flake sizes, while the MoS_2_ nanosheets displayed a flake size of approximately 1 um. Meanwhile, Figure 2c shows a uniform distribution of ZnO nanoparticles. From the SEM image in Figure 2d, we can clearly see that the smaller-sized MoS_2_ nanoplatelets tended to adhere to the surface of larger-sized graphene nanoplatelets to form the heterostructures due to the van der Waals effect. In addition, the heterostructure of ZnO/MoS_2_ can be observed in Figure 2e, in which a large amount of ZnO nanocrystals is distributed on the surface of the MoS_2_. Similarly, the final heterostructures of the ZnO/MoS_2_/graphene ternary composite are shown in Figure 2f, with the uniformly distributed ZnO nanoparticles on the interlayered MoS_2_ and bottom-layered graphene nanoplatelets. To further confirm the existence of every element component, the energy-dispersive X-ray spectroscopy (EDS) was conducted on the samples of the MoS_2_/graphene heterostructure, the ZnO/MoS_2_ heterostructure, and the ZnO/MoS_2_/graphene ternary heterostructure, respectively. The typical element components of each material were measured, which further confirmed the formation and existence of these heterostructures, as shown in Figure 2g,h,i.

### 3.2. Structural Characterization of Materials

**Figure 3:**
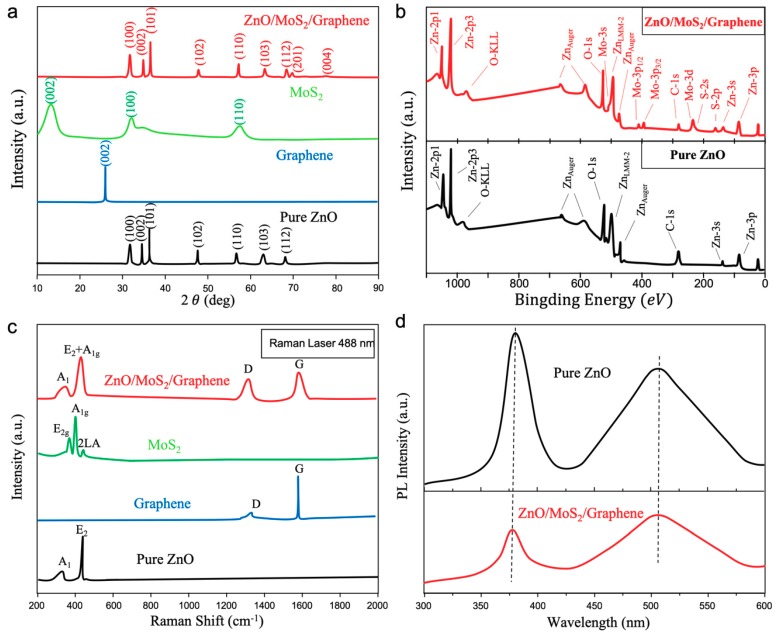
Structural characterization of the various samples. (**a**) XRD measurements of each component; (**b**) X-ray photoelectron spectrometer (XPS) data of pure ZnO and ZnO/MoS_2_/graphene heterostructures; (**c**) Raman characterizations of each component; (**d**) photoluminescence (PL) spectra of the ZnO and ZnO/MoS_2_/graphene heterostructures.

The structural analysis of the various items and their components was performed using XRD, XPS, Raman, and PL spectroscopy. Figure 3a shows the XRD images of ZnO, graphene, MoS_2_, and the ZnO/MoS_2_/graphene ternary heterostructures. For pure ZnO nanocrystals, the diffraction peaks appeared at 2θ = 31.3, 34.6, 36.1, 47.7, 56.2, 62.9, and 67.7; these peaks can be attributed to (100), (002), (101), (102), (110), (103), and (112) lattice planes, respectively. A comparison of the XRD patterns with the (100), (002), (101) and other diffraction peaks of standard ZnO materials demonstrates the existence of a polycrystalline hexagonal wurtzite crystal orientation for ZnO [38]. When we compared the XRD data of pure ZnO with ZnO/MoS_2_/graphene, it was easy to see that the (100), (002), and (101) diffraction peaks had a slight left shift, indicating that the ZnO lattice constant became larger due to the strong interactions of van der Waals forces in the heterostructures. Moreover, with respect to the pure MoS_2_ nanosheets, we compared the diffraction peak positions with the standard card JCPDS 37-1492, which was consistent with our results, proving that the product is a hexagonal phase. Moreover, the presence of the (002) peak indicated a good crystallization of the sample. The XRD diffraction spectrum of graphene nanoplatelets exhibited a standard graphene diffraction peak at 2θ = 26°, which was different from the diffraction peak of graphene oxide at 2θ = 10°. This indicates that there are no damages or defects between the layers of graphene, as proof of its excellence. Regarding the ZnO/MoS_2_/graphene heterostructures, they mainly exhibited the diffraction peak characteristic of ZnO, owing to the fewer components of graphene and MoS_2_ in the composition. 

Further, XPS spectra are shown in Figure 3b, the C-1s peak of 284.3 eV mainly corresponded to the C-C of sp^2^ hybridization in graphene [39]. Furthermore, the peaks observed at 1020 eV and 1045 eV corresponded to the 2p^3/2^ and 2p^1/2^ peaks of Zn-2p, respectively, which was assigned to the ZnO [40]. We can find that, in pure ZnO, the two peaks of 2p^3/2^ and 2p^1/2^ of Zn-2p had a very slight shift. The reason may be that the formation of a heterojunction in the complex that promoted the charge transferring. In addition, we also observed Mo-3d and S-2p core levels at 228.4 eV and 162.1 eV in agreement to previous reports [41,42,43,44]. The High Resolution-XPS results of C-1s, Mo-3d, and S-2p are shown in the Supporting Information (Figure S1, Supporting Information). From the HR-XPS data of Mo-3d core level, we can conclude that there is only a Mo^4+^ chemical state corresponding to MoS_2_. Moreover, considering that the MoS_2_ is prepared by a physical method (electro-assisted liquid-phase exfoliation method), almost no MoO*_x_* exists in the composite, resulting in the lack of another doublet peak of Mo-3d in the XPS result.

To further characterize the quality of materials, the Raman spectra of ZnO, graphene, MoS_2_, and ZnO/MoS_2_/graphene were measured, as shown in Figure 3c, which illustrated the small defect peak of prepared graphene nanoflakes as the superior electron-accepting layers, and the physical prepared multi-layered MoS_2_ nanosheets as the excellent electron-transport-layered materials. However, after combining the graphene nanosheets with other materials to form the heterostructures, the D peak of the graphene in the composite became significantly higher, due to the influence of the internal effect in the heterojunction. Simultaneously, this effect was also found in the ZnO, which is evidenced by the left shift of the ZnO diffraction peaks in the XRD spectra. An explanation for this is that an increase of the lattice constant causes the occurrence of more oxygen-vacancy defects, which further results in an increase of the visible-light absorption capacity of the materials, eventually improving the catalytic efficiency of the composite. In addition, from the Raman spectra of ZnO/MoS_2_/graphene composite, we are able to explain the widening A1 and E2 peaks by the superimposition of the Raman signals from MoS_2_ and pure ZnO nanoparticles. 

Photoluminescence (PL) spectra were also measured on the ZnO nanoparticles and ZnO/MoS_2_/graphene heterostructures at room temperature, as given in Figure 2d, for an excitation wavelength of the PL spectrum of 372 nm. It is evident from the PL spectrum that by combining ZnO with MoS_2_/graphene, the PL emission peak was reduced, indicating less photogenerated electron-hole recombination. The mechanism can be described as follows: The photogenerated electrons are more naturally diffuse into the graphene layer due to the excellent carrier mobility of graphene and the matched energy levels ($E_{c}^{ZnO}>E_{c}^{MoS_{2}}>E_{Fermi}^{graphene}$), resulting in an extension of the excited-carrier lifetime. Taking into account the fact that the existences of ZnO and MoS_2_ increases the specific surface area, the adsorption capacity of the ZnO/MoS_2_/graphene was enhanced and led to a high photocatalytic ability. 

### 3.3. Micromechanism of Enhanced Photocatalytic Effects

**Figure 4:**
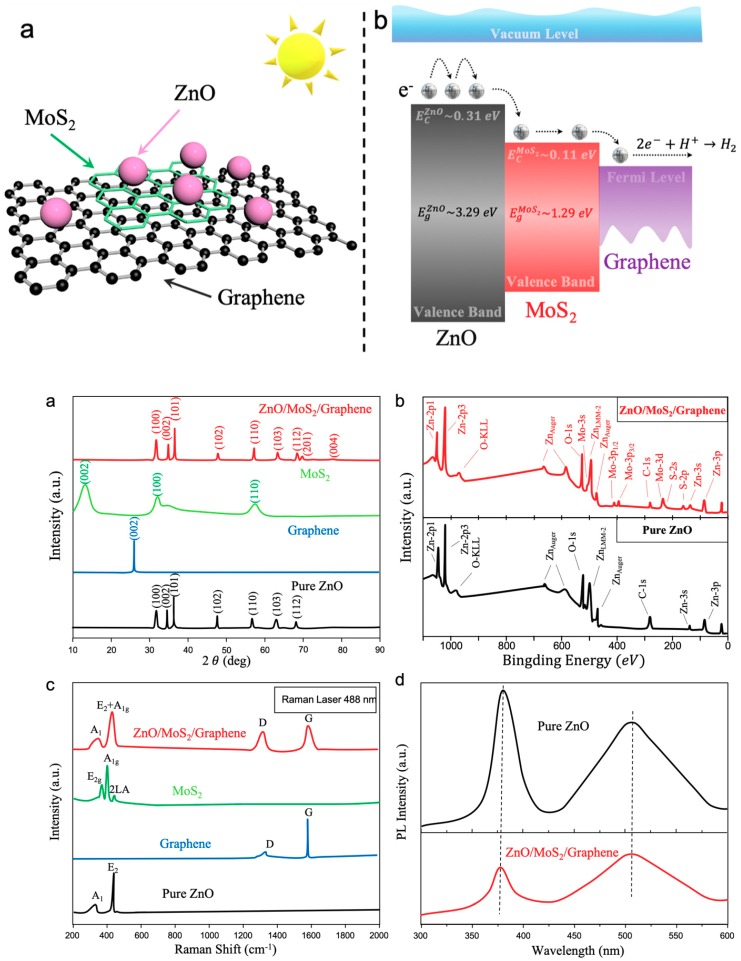
Schematics of heterostructures and electron transfer in photocatalytic processes. (**a**) 3D view of the ZnO/MoS_2_/graphene heterostructures; (**b**) energy diagram and generated electrons transfer during visible-light illumination.

The designed ZnO/MoS_2_/graphene heterostructures and the electron transfer during the photocatalytic processes are illustrated in Figure 4. The primary reasons for the excellent photocatalytic ability of ZnO/MoS_2_/graphene can be summarized as follows: (1) Introducing a MoS_2_ layer increases the specific surface area and inhibits the self-stacking of graphene nanoplatelets, providing more active sites [45,46,47,48]. (2) The energy levels of MoS_2_ and graphene are reasonably matched, enhancing the electron transportation between layered materials; in this way, the possibilities of photogenerated electron-hole recombination are lowered. (3) As a two-dimensional material with higher carrier mobilities, graphene acts as an excellent electron acceptor. (4) The interlayer MoS_2_ serves as a visible-light absorbance material in this structure due to its adequate band gap. The band gaps of multi-layer MoS_2_ nanoplatelets and ZnO nanocrystals are 1.29 eV and 3.29 eV, respectively [49]. According to the previous report, we know that the values of $E_{c}^{Zno}$ and $E_{c}^{MoS_{2}}$ are −0.31 and −0.11 eV, respectively [14]. Considering the fact that $E_{c}^{ZnO}>E_{c}^{MoS_{2}}>E_{Fermi}^{graphene}$, the photogenerated electrons in ZnO are easily transferred to the graphene nanoplatelets through MoS_2_, as shown in Figure 4b. Moreover, due to the existence of interlayered MoS_2_, electrons can travel faster, which reduces the possibility of the recombination of electrons and holes [50,51]. Lastly, the electron-storage capacity of pure graphene nanoplatelets is superior to that of reduced graphene oxide or graphene oxide, because the nanoplatelets do not bond with any functional group, resulting in a higher photocatalytic ability.

### 3.4. Photocatalytic Measurements

**Figure 5:**
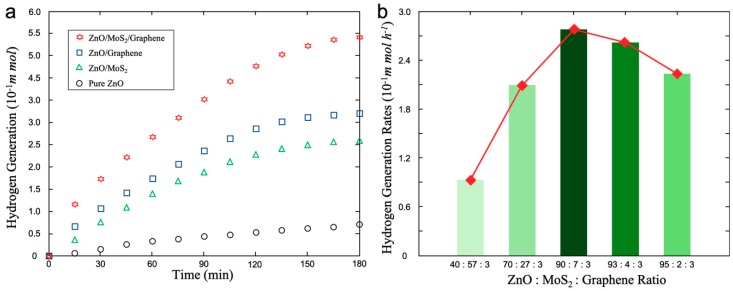
Photocatalytic efficiency measurements. (**a**) Diagram of hydrogen generation rates of ZnO, ZnO/graphene, ZnO/MoS_2_, and ZnO/MoS_2_/graphene catalysts; (**b**) hydrogen generation rates diagram of various componential ZnO/MoS_2_/graphene photocatalysts.

The photocatalytic activity was evaluated by measuring the ability of hydrogen generation under visible-light illumination in various photocatalysts. Figure 5a compares the results of the photocatalytic abilities of four nanomaterials, ZnO, ZnO/graphene, ZnO/MoS_2_, and ZnO/MoS_2_/graphene. The hydrogen generation rate is nearly zero with the ZnO photocatalyst only, whereas ZnO/MoS_2_/graphene leads to maximum hydrogen generation. Due to the synergistic effect of MoS_2_ and graphene nanoplatelets, the recombination rate of photogenerated electron-hole pairs is strongly reduced. Figure 5b shows the differences between these five types of components regarding photocatalytic efficiency. From this diagram we know that the maximum efficiency is achieved with ~ 90% ZnO, ~ 7% MoS_2_, and ~ 3% graphene. This phenomenon can be explained by the various roles played by ZnO, MoS_2_, and graphene in the catalytic process. A sufficient amount of ZnO nanoparticles provided a large number of photogenerated electrons, leading to an increase of the hydrogen generation rates of the photocatalysts [52,53,54]. We also notice that the self-limiting effect exists in the photocatalytic process because the catalyst itself hinders the catalytic reaction. 

In comparison with separate studies referenced in this paper, the ZnO/MoS_2_ photocatalyst prepared by Yuan et al. has a catalytic efficiency of about 0.8 mmol h^−1^ g^−1^ [46]; they also prepared a MoS_2_-graphene/ZnIn_2_S_4_ composite with a catalytic efficiency of about 4.2 mmol h^−1^ g^−1^ [49]. Bhirud et al. prepared N–ZnO/graphene nanocomposites, whose catalytic efficiency reached about 5 mmol h^−1^ g^−1^ [55]. Kumar et al. prepared a ZnO–MoS_2_–Reduced Graphene Oxide composite with a catalytic efficiency of 28.6 mmol h^−1^ g^−1^ [14]. In this work, we prepared a ZnO/MoS_2_/graphene composite, whose catalytic efficiency can reach 5.4 mmol h^−1^ g^−1^ (we measured 50 mg photocatalyst and converted to the same units). As a comparison, we can conclude that this work provides a high-throughput method for preparing higher-efficiency photocatalytic materials, which has a driving role in the application of photocatalysis in industry.

## 4. Conclusions

In this study, we presented a novel high-efficient photocatalyst based on the ZnO/MoS_2_/graphene sandwiched heterostructures. This new material was synthesized by taking advantage of the high electron densities of ZnO nanoparticles, the ultra-high electron mobilities and specific surface area of graphene nanoplatelets, and the enhanced visible-light absorption properties of MoS_2_, which allowed a highly efficient production of hydrogen by ZnO/MoS_2_/graphene enabled photocatalysis. Also, the production of these materials occurred mainly through chemical and liquid-phase exfoliations, which are suitable for large-scale industrial production. In summary, this work combined the excellent properties of zero-dimensional nanoparticles and two-dimensional nanomaterials, developed a novel layer-by-layer nanostructured photocatalyst, and eventually realized a highly efficient hydrogen production under sunlight. It opens new avenues for the high-throughput production of superior hydrogen-evolution photocatalysts that are of significant importance to a clean energy revolution. 

## Supplementary Materials

The following are available online at https://www.mdpi.com/1996-1944/12/14/2233/s1. Figure S1. HR XPS of C-1s, Mo-3d and S-2p after reducing noisy.
