# Supplementary material for: High-throughput Production of ZnO-MoS2-Graphene Heterostructures for Highly Efficient Photocatalytic Hydrogen Evolution
Source: Materials (Basel). 2019 Jul 11;12(14):2233. doi: 10.3390/ma12142233 (PMC6678946; doi:10.3390/ma12142233)
Supplement: Supplementary file 1 [file materials-12-02233-s001.pdf]

materials

MDPI

Supporting Information

High-throughput Production of ZnO-MoS_2-Graphene Heterostructures for Highly Efficient Photocatalytic Hydrogen Evolution

Haocong Dong [1,2], Junzhu Li [2,3], Mingguang Chen [3], Hongwei Wang [2,4], Xiaochuan Jiang [2,5], Yongguang Xiao [1,*], Bo Tian [2,3,*] and Xixiang Zhang [3,*]

[1] Key Laboratory of Film Materials & Application for Equipment, School of Materials Science and Engineering, Xiangtan University, Xiangtan 411105, China; haocong.dong@11dlab.com (H.D.)

[2] Eleven-Dimensional Nanomaterial Research Institute, Xiamen 361000, China; junzhu.li@kaust.edu.sa (J.L.); hongwei.wang@11dlab.com (H.W.); xiaochuan.jiang@11dlab.com (X.J.)

[3] Physical Science and Engineering Division, King Abdullah University of Science and Technology (KAUST), Thuwal 23955-6900, Saudi Arabia; mingguang.chen@kaust.edu.sa (M.C.)

[4] State Key Laboratory of Advanced Optical Communication Systems and Networks, Department of Electronic Engineering Shanghai Jiao Tong University, Shanghai 200240, China

[5] Department of Astronomy, Xiamen University, Xiamen 361000, China

* Correspondence: bo.tian@kaust.edu.sa (B.T.); ygxiao@xtu.edu.cn (Y.X.); xixiang.zhang@kaust.edu.sa (X.Z.)

We provided a survey XPS in the main text to confirm the existence of each element component. In addition, the HR-XPS results are shown as follows:

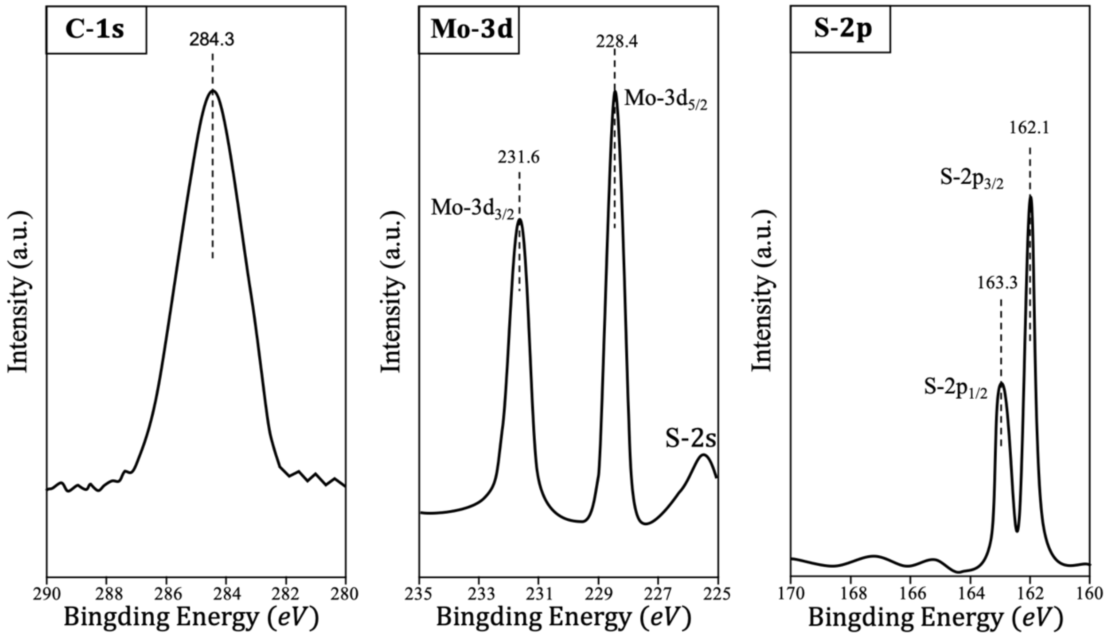

Figure S1. HR XPS of C-1s, Mo-3d and S-2p after reducing noisy.
